# Fractionation of Mastic Gum in Relation to Antimicrobial Activity

**DOI:** 10.3390/ph2010002

**Published:** 2009-04-01

**Authors:** Mohammad Sharif Sharifi, Stuart Loyd Hazell

**Affiliations:** 1 School of Medicine, University of Western Sydney, Australia; 2 Faculty of Sciences, University of Queensland, Australia; Email: SHazell@fusidium.com

**Keywords:** Mastic Gum, *Helicobacter pylori*, *Pistachia lentiscus.*

## Abstract

Mastic gum is a viscous light-green liquid obtained from the bark of *Pistacia lentiscus var. chia.* which belongs to the *Anacardiaceae* family. The gum has been fractionated to investigate the antimicrobial activity of the whole gum and its fractions against various strains of *Helicobacter pylori*. The polymeric gum fraction was separated from the essential oil and the resin (trunk exudates without essential oil) to assess and compare the anti-*H. pylori* activity of the polymer fraction against lower molecular weight fractions, the gum itself and masticated gum. The polymer fraction was also oxidized and assessed for antimicrobial activity.

## 1. Introduction

Various fractions of mastic gum were isolated by steam distillation, solvent extraction and column chromatography, and then tested against *H. pylori* by determining Minimum Inhibitory Concentration (MIC) and kill kinetics. Oxidative modification of the polymer fraction was carried out to investigate any change in biological activity. The *H. pylori* strains 26695, J99, RSB6, P10, SS1, SS2000, N6, NCTC11637 and RU1 were used for the MIC determinations and strain 26695 was used for the kill kinetics.

## 2. Results and Discussion

The MIC values of the fractions ranged from 125 to 1,000 μg/mL. The most active fraction was found to be the polymer and its activity was enhanced by oxidation, being twice the original activity. The activity of the masticated gum was also twice that of the original gum.

### 2.1. Introduction

Historically, trunk exudates of *Pistachia lentiscus* (mastic gum) have been used for the treatment of stomach ulcers. Archaeologists in 1982 found a late Bronze Age shipwreck with 100 jars filled with mastic that had been used by the Egyptians for medicinal purposes [1]. The ancient Greek physicians Galenus in “Simpliciun medicamemtorum temperamentis ac faculatibus libri XI” and Dioscorides in “De Materia Medica” have described the properties and usage of mastic oil. The Persian pharmacist, physician and philosopher Avicenna (980-1037) prescribed mastic gum for abdominal pain, heartburn and topological infections. The Arab physician Ibn Al-Baytar, living in the 13^th^ century, prescribed mastic gum for upper abdominal pain, heartburn, gastric and intestinal ulcers [2]. 

The genus *Pistachia* from the *Anacardiaceae* family consists of eleven species of trees found in some Mediterranean countries and in Southern and Central America [3]. Substantial work has been done on characterization of chemical composition of *Pistachia lentiscus* and some other species which are widely spread around the Mediterranean countries as well as the Zagros Mountains and particularly in western and northern Iran and eastern and northern Iraq [4].

Several studies have been carried out on the chemical composition of the essential oils of *P. lentiscus.* The main constituents are monoterpenes [3,4,5,6,7]. In 1954 Barton and Seoane fractionated mastic gum into two major fractions, an acidic and a neutral fraction [8], and, in 1998, Van Den Berg *et al*. [9] identified an unusual polymer constituent, “1,4-poly-*β*-myrcene”.

The aqueous extract of *P. lentiscus* var. *caia.* has been investigated for antifungal activity against *Microsporum canis, Trichophyton mentagrophytes* and *Trichophyton violaceum* [10]. The extract reduced the growth of colonies by 36-100% [10] A double blind controlled clinical trial showed mastic gum to have a significant effect in relieving the symptoms and in healing of duodenal ulcers [11,12].

The antimicrobial activity of mastic gum has also been evaluated against clinical isolates of *H. pylori* and it was shown to have an MIC value of 125 µg/mL [13]. However, only the whole mastic gum has been used in these studies. An abstract was also published, in which the antibacterial activity of mastic fractions were briefly mentioned [14]. The extracts and major composition of mastic gum were tested for their antimicrobial activity against *H. pylori* [15].

In this study, we have examined the anti-*H. pylori* activity of individual fractions of mastic gum together with whole gum, and the masticated form of the gum. We have also investigated the activity of the highest molecular weight fraction following its aerial oxidation.

## 3. Material and Methods

### 3.1. Extraction of Essential Oils and Resin

The isolation of the essential oils from a sample of crude gum, purchased from Sigma-Aldrich (Sydney, Australia), was achieved by steam distillation with water cohobation of the gum (50 g) in a Dean and Stark apparatus, which was modified to give lower phase return of the water. The steam distillation was carried out over 10 h. Approximately 5 mL of pentane was then added to the distilled oil to increase its volume. The pentane and oil solution was dried using anhydrous sodium sulfate (2 g) and, after decanting and filtering to remove the sodium sulfate, the solution was transferred to another container and the pentane allowed to evaporate in air overnight. The essential oil from the gum (2.6 g) was then retained for further analysis and for anti-microbial activity trials against *H. pylori.* The crude resin (42.5 g) remaining after steam distillation was then fractionated.

### 3.2. Fractionation of the Resin

The finely powdered crude resin (42.5 g) was dissolved in anhydrous diethyl ether (50 mL), diluted with anhydrous methanol (350 mL), and allowed to stand in a stoppered flask overnight. After decanting from any insoluble residue (0.08 g), the solution was evaporated under vacuum. The resulting solid was dissolved in anhydrous diethyl ether (50 mL), diluted with anhydrous methanol (350 mL), allowed to stand, and then decanted from any insoluble residue (0.89 g). After evaporating the supernatant solution under vacuum, the solid product was allowed to dry in air overnight. The dry residue was accurately weighed (41.1 g), dissolved in anhydrous diethyl ether (50 mL), diluted with anhydrous methanol (350 mL) and then to this solution milli-Q water (40 mL) was gradually added followed by 3 g of magnesium chloride. The solution was mixed thoroughly and allowed to stand overnight. A yellow viscous liquid was formed in the aqueous layer. The precipitate that had formed was decanted and designated as ‘Polymer Fraction’ (9 g). 

The ethereal layer was dried over anhydrous sodium sulphate, filtered, evaporated and the resulting solid product accurately weighed (32.5 g). This product was then dissolved in anhydrous diethyl ether (50 mL) and diluted with anhydrous methanol (300 mL). The resulting solution was extracted with sodium carbonate solution 5% (w/v, 100mL) and the sodium carbonate extract acidified and then re-extracted with fresh anhydrous diethyl ether (100 mL). This ethereal solution was evaporated under vacuum and a sodium carbonate acidic fraction (11.71g, ‘fraction a’), so thus obtained.

The original ether solution was then extracted with 0.5 N sodium hydroxide (70 mL), producing a viscous liquid above the aqueous layer, a light yellow aqueous solution, and an oily precipitate. The oily precipitate was collected in a stoppered bottle for further analysis. The viscous liquid and light yellow solution were individually acidified, re-extracted into fresh ether and the ethereal extracts were dried over anhydrous sodium sulphate, filtered and evaporated under vacuum. There were thus obtained a sodium hydroxide-(in) soluble acid fraction from the viscous liquid, ‘fraction b’ (5 g), a second sodium hydroxide-soluble acid fraction, ‘fraction c’ (2 g), and a sodium hydroxide-reactive insoluble oily precipitate fraction, ‘fraction d’ (0.9 g). The original ethereal layer was dried over anhydrous sodium sulphate, filtered and then evaporated under vacuum, giving the ‘neutral fraction’ (12.5 g). 

The polymer fraction (9 g) was dissolved in methanol (150 mL) and added to a chromatography column (600 mm x 50 mm internal diameter) packed with Sephadex 200G and eluted with methanol, under gravity pressure at an approximate rate of 200 µL/minute. A total of fourteen 20 mL light yellow fractions, two pale yellow fractions, and two colorless fractions were collected. The first fraction eluted (17 mL), containing the highest molecular weight components, classified as the High Molecular Weight Components. To this fraction were added 3 mL of milli Q water and 0.1 g of magnesium chloride. A white precipitate was formed, which was isolated by decanting the aqueous layer and any remaining moisture was removed by freeze drying at –40° C (2.1 g) and the sample then retained for further analysis. The molecular weight distribution of the polymer was determined by Gel Permeation Chromatography (GPC).

### 3.3. Mastication

A 2 g sample of the mastic gum resin was masticated for 4 h., then washed with distilled water and dried in a desicater (2.14 g). A portion of the dried masticated resin (50 mg) was then dissolved in methanol (2 mL) and a level of oxidation confirmed by adding a few drops of the solution to 1 mL of 2,4- dinitrophenylhydrazine (2,4-DNP) reagent. A similar test was carried out on unmasticated resin.

### 3.4. Oxidation of the Polymer Fraction

A sample of the polymer fraction (50 mg) was dissolved in methanol (2 mL) and tested for the presence of carbonyl groups by adding a few drops of the solution to 1 mL of 2,4-DNP reagent. Finely powdered polymeric fraction (1 g) was suspended in 50 mL. of distilled water and analytical grade air bubbled through the suspension for 24 h. The solid was filtered off and allowed to air dry (1.11 g). The aerated, dried polymer (50 mg) was then dissolved in methanol (2 mL.) and oxidation confirmed by adding a few drops of the solution to 1 mL of 2,4-DNP reagent. 

### 3.5. Bacterial Strains and Culture Conditions

*H. pylori* strains 26695, J99, RSB6, P10, SS1, SS2000, N6, NCTC11637 and RU1 were obtained from the culture collection of the University of New South Wales and were used for the MIC determinations. Strain 26695 was used for kill studies. The strains were grown on Blood Agar Base No. 2 (Oxoid, Basenstoke, UK) supplemented with 7% defibrinated horse blood, amphotericin B (2μg/mL) and Skirrow’s selective supplement, consisting of the antibiotics Trimethoprim (5 μg/mL), Polymixin B (2.5 μg/mL), and Vancomycin (10 μg/mL). Bacterial cultures were incubated in an atmosphere of 10% CO_2_ in air, 95% relative humidity at 37° C.

### 3.6. Minimum Inhibitory Concentration

Stocks of 100 mg/mL of mastic in methanol were prepared and used to determine the Minimum Inhibitory Concentration and also to investigate kill kinetics. To sterile molten blood agar base (184 mL at 55ºC), 2 mL of mastic solution from the stock was added, mixed using an ultrasonic bath and allowed to stand overnight at 55ºC. Then 14 mL of defibrinated horse blood (Oxoid UK) was added to give a concentration of 1 mg of mastic in 1 mL of blood agar. Six serial dilutions were then performed by diluting the above blood agar containing mastic with freshly prepared blood agar, giving mastic concentrations of 1.0 mg/mL, 0.5 mg/mL, 250 μg/mL, 125 μg/mL, 62.5 μg/mL and 31.25 μg/mL. Plates were poured from each dilution, and were inoculated with the various *H. pylori* strains. The presence or absence of growth was noted after five days of incubation at 37ºC, in a Stericult incubator (Forma Scientific, USA) at 37°C with 95% relative humidity and 10% CO_2_ to reduce oxygen and provide microaerophillic environment with each test being performed in duplicate. 

For all MIC tests 10 μL/mL, 5 μL/mL, 2.5 μL/mL, 1.25 μL/mL, 0.62 μL/mL or 0.32 μL/mL methanol in agar base was used as control. The MIC was determined as the lowest concentration of the mastic that showed no growth. The same procedure was subsequently performed using the resin, masticated resin, essential oils of the mastic, the polymer and oxidised polymer fractions, the acidic fractions ‘a’, ‘b’ and ‘c’, the oily fraction ‘d’, the neutral fractions, and all other 17 lower molecular weight fractions which were isolated from the polymer fraction. 

### 3.7. Kill Kinetics

Time-kill kinetic experiments were performed with static liquid cultures containing Isosensitest broth (Oxoid) supplemented with 5% horse serum (Oxoid), 4.2 mg/mL Amphotericin B and Skirrow’s selective supplement. The inoculum was harvested with Isosensitest broth from 36 hr cultures grown on Campylobacter Selective Agar (CSA). To ensure that the strain had the same concentration, the Optical Density 600 (OD600) of the inoculum was adjusted to 0.1 and diluted in the culture medium to give a starting concentration of 1.00 x 1.00 E + 07 at 600 nm. Each culture was incubated for 2 h. to allow recovery of the bacteria before unoxidised or oxidized polymer were added at their respective MIC and 5MIC concentrations. Control cultures at MIC and 5MIC containing methanol were also performed. Samples were taken at 0 to 120 min at 5 min intervals, diluted with 0.9% saline, plated on *Campylobacter* Selective Agar (CSA) using a spiral platter and incubated at 37°C in a CO_2_ incubator for 48h. to determine viable colonies.

## 4. Results

### 4.1. Fractionation

**Figure 1:**
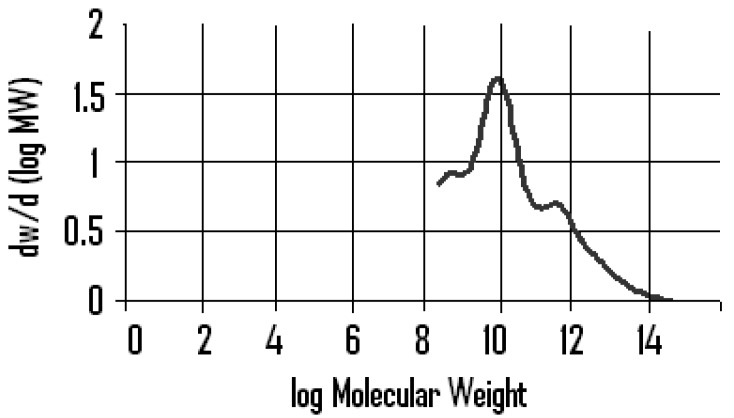
Molecular weight distribution within the polymeric fraction of "mastic gum" (Mn 80,000, Mw above 120,000).

The yield of essential oil obtained was 5.20% (w/w) of the mastic. The polymer fraction was found to have a number average molecular weight of 80,000 and a weight average molecular weight of above 120,000 (Figure 1) by Gel Permeation Chromatography (GPC), and was 4.2% (w/w) of the mastic gum. No change in color was observed when it was treated with 2,4-DNP while a yellow precipitated formed with the aerated sample. A yellow precipitate was also formed when masticated mastic was treated with 2,4-DNP, but no change in color was observed when mastic was treated with 2,4-DNP. The yield of acidic fraction from the sodium carbonate extract was 23.42% (w/w) of the mastic and the acidic fractions (‘b’ and ‘c’) from the sodium hydroxide extract was 14.0 % (w/w) of the mastic. The yield of oily fraction was 1.8% (w/w). The neutral fraction yielded 25.0% (w/w) of the mastic gum.

### 4.2. Antibacterial Activity

The MIC values obtained were dependent upon the *H. pylori* strain used and were found to range from 250 to 1,000 μg/mL for the polymer and from 125 to 500 μg/mL for the oxidized polymer. For the whole mastic the range was 750-1,000 μg/mL, for the resin 500-750 μg/mL, for the masticated resin 500 mg/mL, for acidic fraction ‘a’ 750 mg/mL, for acidic fraction ‘b’ 750-1,000 mg/mL, for acidic fraction ‘c’ 750-1,000 mg/mL, and for the essential oil 500-1,000 mg/mL (Table 1). The neutral fraction had no activity at the concentrations tested. Similarly, fractions 2-18 from the polymer showed no activity.

**Table 1:** MIC Values with nine strains of *Helicobacter pylori* for Fractions of mastic gum.

Bacterial Strains	Polymer (µg/mL)	Oxidized polymer (µg/mL)	Fractions 2-18 (µg/mL)	Mastic (µg/mL)	The Resin (µg/mL)	Masticated Resin (µg/mL)	Acidic Fraction (µg/mL)			Neutral Fraction (µg/mL)	Essential Oils (µg/mL)
							a	b	c		
26695	500	250	NA	750	750	500	750	1000	1000	NA	1000
J99	250	125	NA	750	750	500	750	1000	1000	NA	1000
RSB6	1000	500	NA	750	750	500	750	1000	1000	NA	1000
P10	500	250	NA	750	750	500	750	1000	1000	NA	1000
SS1	1000	500	NA	500	500	500	750	750	750	NA	500
SS2000	1000	500	NA	500	500	500	750	750	750	NA	500
N6	250	125	NA	750	750	500	750	1000	1000	NA	1000
NCTC 11637	250	125	NA	750	750	500	750	1000	1000	NA	1000
RU1	250	125	NA	750	750	500	750	1000	1000	NA	1000

NA: No Activity

**Figure 2:**
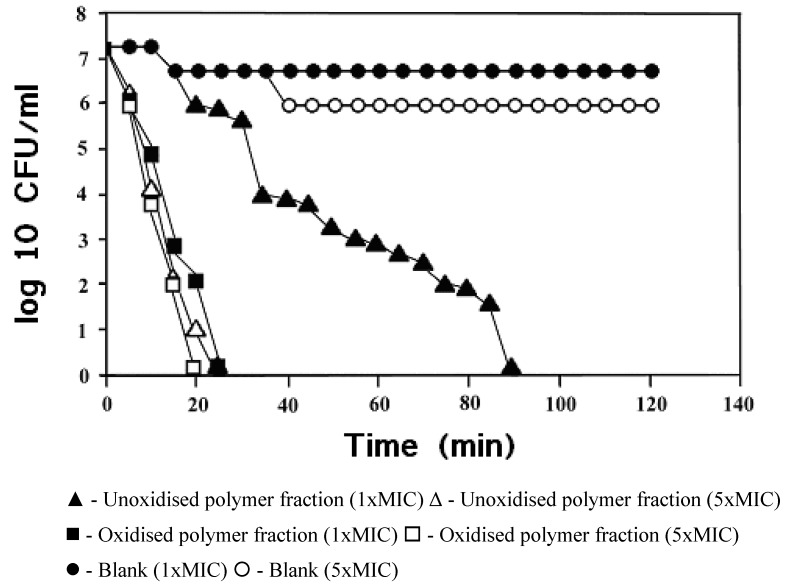
Kill kinetics of *H. pylori* strain 26695 in the presence of polymer fraction and oxidised polymer fraction at their MIC and at 5 times their MIC.

MIC values were also calculated as activity (log 1/C in mg/mL) and the average activity across all strains for each material under test, relative to the activity shown by whole mastic. The average relative activities demonstrated, in descending order, were: oxidized polymer (3.87), polymer (2.04), masticated resin (1.83), unmasticated resin (1.00), whole mastic (1.00 by definition), acidic fraction ‘a’ (0.76), essential oils (0.41), acidic fractions ‘b’ and ‘c’ (0.16), neutral fraction (0.00) and fractions 2-18 of the polymer (0.00). The polymer fraction killed 99% of *H. pylori* strain 26695 in 90 min. at its MIC concentration (0.5mg/mL) while the oxidized polymer killed 99% of this strain in 25 min. at its MIC concentration (0.25mg/mL) (Figure 2). 

## 5. Discussion

Mastic gum has a history of informal use in anti-ulcer therapy spanning at least ten centuries [2]. More recently, mastic gum from *P. lentiscus* has shown to have *in vitro* antimicrobial activity against *H. pylori* [13,14,15,16,17] and to be effective against duodenal ulcers in clinical trials [11,12]. Although not reported prior to this study with *H. pylori*, antimicrobial activity against a number of microorganisms has been demonstrated *in vitro* by various components of the essential oil of the gum. Despite the fact that a number of other plants incorporate these components at various levels in their essential oils, their comparable historical use or advocacy in anti-ulcer therapy has not been reported. The question arises therefore as to why mastic gum and some other species of *Pistacia* might seemingly be so singularly and historically advocated for this purpose.

There are three more obvious aspects by which mastic gum might differ from other plants in this regard. It has been shown to incorporate (1) an unusual polymer, polymyrcene [9], and (2) a range of characteristic di- and tri-terpenes and –terpenoids [8] as part of its components, and (3) it is masticated in use, a practice consistent with its physical form and properties. The last suggests a possible *in situ* oxidation of the gum, taking place in therapeutic use [4].

This study has therefore sought to (1) fractionate the gum and investigate any anti-*H. pylori* activity of its various major fractions; (2) isolate the highest molecular weight fraction of the gum and investigate the activity of this fraction; and (3) investigate the possibility of a potential increase in activity of the gum as a consequence of its mastication.

The results show that the greatest level of activity (twice that of the gum itself, Table 1) is demonstrated by the components of the highest molecular weight fraction (4.2% by weight of the gum). This fraction incorporates a wide range of molecular weights (Figure 1) from approx. 200 (possibly di-terpenes/terpenoids) to above 50,000 (high molecular weight polymeric components) with a number average molecular weight of the polymer fraction being 53584.

Some activity is also shown by those components of the resin which demonstrate strong acid properties (i.e. the sodium carbonate soluble fraction, 23.42% of the gum by weight) although, like all other fractions, these acidic components show a lower level of activity than the whole mastic itself. Significantly lower levels of activity are demonstrated by the remaining weak (sodium hydroxide soluble) acid fractions (14% by weight) and essential oils (5.2% by weight), whilst no detectable activity was detected at the concentration levels tested for any of the other components, including the neutral components (25% by weight) of the resin.

Mastication of the resin was shown to result in the introduction of aldehyde and/or ketone structures within it, consistent with an oxidation process having taken place. It also resulted in a significant increase in its anti-*H. pylori* activity (Table 1). Similarly, exposure of the polymer fraction to oxygen resulted in both the incorporation of aldehyde/ketone structures and a significant increase in activity. In each case, exposure to oxygen resulted in an effective doubling of anti-*H. pylori* activity.

This enhanced activity was confirmed by the kill kinetics studies carried out on the polymer fraction, this fraction, in both oxidised and unoxidised form (see Figure 2). At its MIC concentration of 250 μg/ml this oxidised fraction killed 99% of strain 26695 *Helicobactor pylori* within 25 mins, whilst the unoxidised fraction, even at its MIC concentration of 500 μg/ml, required 90 min to reach a comparable 99% kill level.

These results are consistent with the concept that the anti-*Helicobactor pylori* activity of the components of mastic increase as their polarity increases, and in particular as a consequence of the presence of aldehyde and/or carboxylic acid groups within their structures, the level of these groups potentially increasing as a consequence of oxidation *in situ*. They are also consistent with higher activity being demonstrated by higher molecular weight components, including oxidised polymeric structures.
